# The Fate of Childhood Memories: Children Postdated Their Earliest Memories as They Grew Older

**DOI:** 10.3389/fpsyg.2015.02038

**Published:** 2016-01-12

**Authors:** Qi Wang, Carole Peterson

**Affiliations:** ^1^Department of Human Development, Cornell University, IthacaNY, USA; ^2^Department of Psychology, Memorial University of Newfoundland, St. John’sNF, Canada

**Keywords:** childhood amnesia, postdating, earliest memory, memory age estimate, prospective study, telescoping

## Abstract

Childhood amnesia has been attributed to the inaccessibility of early memories as children grow older. We propose that systematic biases in the age estimates of memories may play a role. A group of 4- to 9-year-olds children were followed for 8 years, recalling and dating their earliest childhood memories at three time points. Although children retained many of the memories over time, their age estimates of these memories shifted forward in time, to later ages. The magnitude of postdating was especially sizable for earlier memories and younger children such that some memories were dated more than a year later than originally. As a result, the boundary of childhood amnesia increased with age. These findings shed light on childhood amnesia and the fate of early memories. They further suggest that generally accepted estimates for people’s age of earliest memory may be wrong, which has far-reaching implications.

## Introduction

Most people can remember their childhood experiences from about 3 or 4 years of age but not earlier, a phenomenon commonly termed childhood amnesia (Pillemer and White, 1989; Bauer, 2007; Peterson, 2012). Although retrospective research on adults’ earliest childhood memories is abundant, prospective research on children’s earliest childhood memories is relatively scarce. The developmental data are critical, however, in unraveling the mechanisms that produce childhood amnesia and further identifying factors responsible for early memory development. In particular, what happened to the memories for events that occurred in the first years of life?

One commonly held theoretical view is that early memories are *destined* to become inaccessible or forgotten as children get older and that this eventually results in childhood amnesia (Bauer, 2007; Peterson, 2012). In support of this view, cross-sectional studies of children’s childhood recollections have observed an increase in the age of earliest memory with age, whereby older children and adolescents recall their earliest memories from later ages than do younger children (Peterson et al., 2005, 2009; Jack et al., 2009; Tustin and Hayne, 2010). Existing prospective research has also shown that early memories of children exhibit a constant rate of forgetting characterized by the exponential function, which results in a shrinking pool of memories available for later retrieval (Bauer and Larkina, 2014).

However, not all early memories are lost to recollection over the course of development. Given that much of the memory faculty has been in place by preschool age and that young preschoolers are often able to recall events occurring months or even years ago (Nelson and Fivush, 2004; Bauer, 2007; Peterson, 2012), it is possible that some of the early memories may remain accessible as children grow older. Indeed, Peterson et al. (2011) observed in a longitudinal study of earliest memories that 43.6% of preschool through teenage children produced overlapping memories between two interviews spanning across a 2-year period. This finding is critical: it suggests that childhood amnesia may not be a mere result of an obscured period of life and that there may be other explanations.

To explore the possibility, Wang and Peterson (2014) conducted two prospective studies in which they asked 4- to 13-year-olds children to recall and date their earliest memories at two time points, with a 1-year or 2-year interval. Consistent with the earlier observation (Peterson et al., 2011), they found that many memories remained accessible over time. However, children postdated these memories to significantly older ages as time went by, especially memories from earlier years of life. Thus, although children continued to remember many of the same events as their earliest memories, the location in time of the memories shifted to an older age. Wang and Peterson (2014) suggest that this may eventually result in a period of childhood “amnesia” from which no memories are dated, instead of no memories able to be recalled.

These findings are in line with general research on memory dating. Studies have shown that when people recall and date distant memories from their lives, they often make *telescoping* errors: They postdate the memories as if the events have happened more recently than they actually have, which resembles the situation where an object appears closer in distance when viewed through a telescope (Loftus and Marburger, 1983; Rubin and Baddeley, 1989; Janssen et al., 2006). Telescoping has been explained in terms of the smaller or less complete retention for distant memories, which are then dated with less precision than more recent events (Huttenlocher et al., 1988; Rubin and Baddeley, 1989). Conceivably, childhood memories may be particularly prone to telescoping errors given their decreased retention with elapsed time (Pillemer and White, 1989; Bauer, 2007), and children may be particularly vulnerable to telescoping errors due to their limited knowledge of time and memory dating strategies (Friedman, 2005; Wang et al., 2010; Pathman et al., 2013; Pathman and Ghetti, 2014). Although studies with different age groups have demonstrated the malleability of earliest memories (Wang et al., 2004, 2010; Wang, 2006; Peterson et al., 2011; Kingo et al., 2013), the studies by Wang and Peterson (2014) are the first to identify systematic telescoping errors over time in the dating of earliest childhood memories.

Thus, the prospective studies by Wang and Peterson (2014) provide the initial evidence for an alternative explanation for childhood amnesia. Nevertheless, given the limited 1-year and 2-year intervals between interviews, the findings are inconclusive. Will children continue to remember and postdate their memories after a prolonged period of time? Will the memory age estimates become stabilized at some point over the course of development? We investigated these intriguing questions in the present study. In the sample of 125 children of Wang and Peterson (2014), we were able to locate 37 children 8 years later after the initial interview. Thus, we were able to follow this small group of 4–5, 6–7, and 8–9-year-olds children for 8 years, examining their recall and dating of their earliest memories at three time points: an initial interview, a 2-year follow-up, and an 8-year follow-up. We expected that children would continue postdating their memories with elapsed time. On the other hand, we expected that as children grew older, memory age estimates might become part of their memory or personal “knowledge” (e.g., “I was three and a half when my parents took me to Paris the first time”) and thus stabilized. This, coupled with increasing memory retention and memory dating strategies (Friedman, 2005; Bauer, 2007; Pathman et al., 2013; Pathman and Ghetti, 2014), might result in a decrease in the magnitude of postdating among older children and for older memories.

## Materials and Methods

### Ethical Statement

The Interdisciplinary Committee on Ethics in Human Research, Memorial University of Newfoundland, Canada approved the study. Parents were asked if they would give permission for their children to participate, and children were asked to give informed assent.

### Participants

The sample consisted of 37 children who were interviewed three times about their earliest memories over the course of 8 years. At the initial interview, the children included 13 4- to 5-year-olds (seven girls, *M* = 5.04 years, *SD* = 0.66; referred to as the “youngest group” hereafter), 12 6- to 7-year-olds (three girls, *M* = 6.88 years, *SD* = 0.66; referred to as the “middle group”), 12 8- to 9-year-olds (five girls, *M* = 8.94 years, *SD* = 0.48; referred to as the “oldest group”). At the 2-year follow up, the mean age was 7.80, 9.08, and 11.26 years (*SDs* = 0.93, 0.77, and 0.48) for the youngest, middle, and oldest groups, respectively. At the 8-year follow up, the mean age was 14.34, 15.60, and 16.17 (*SDs* = 1.52, 2.23, and 1.16) for the three groups, respectively. The children were from primarily White, middle-class families in Newfoundland, Canada and were part of a larger study investigating children’s memory development. Parents gave permission for their children to participate and children gave informed assent.

### Procedure

A female experimenter interviewed children at home. She asked children to think of their three earliest memories. General prompts such as “What else do you remember about that?” were used to probe children to give as much information as possible. Following each memory, children were asked how old they were when the memory event took place, followed by questions to help them narrow down their age estimate into a particular month or small range of months: “How old were you when this happened?” “Do you remember what time of year it was?” “Was it summer or winter?” “Was it near your birthday/Christmas/Halloween?” If children specified a range of months (e.g., “The summer when I was 3”), the midpoint of that range was used.

Two years after the initial interview, children were interviewed again in an identical procedure during which their three earliest memories were elicited. Children first recalled three memories spontaneously, which yielded a mixture of “initial” (i.e., memories recalled at the initial interview) and “new” memories (i.e., memories recalled for the first time at the 2-year interview). To facilitate children’s recall, a cued-recall procedure followed if children failed to spontaneously produce any of the three “initial” memories they recalled 2 years previously. A synopsis of each of the memories was read to them that contained critical information about the event (e.g., “One time someone tripped you at school and you broke the pot that you had just made.”). After each memory was read, children were asked whether this memory ever happened to them and, if they recognized the memory, they were asked to recall and date the memory. To ensure that children were not simply confirming the cued events, three synopses of “lure” events (i.e., memories recalled by other children) were also read to them. Children invariably identified lures as having never happened to them.

Then, 8 years after the initial interview children were interviewed again identically to their prior interviews. They were first asked to recall and date their three earliest memories. If they failed to spontaneously produce any of the “initial” or “new” memories, a cued-recall procedure followed as in the 2-year interview.

## Results and Discussion

Among the 37 children, 32 (86.5%; nine from the youngest group, 11 from the middle group, and 12 from the oldest group) recalled and dated at least one “initial” memory at both the 2-year and 8-year interviews (*N* = 29) or at one of the interviews (*N* = 3). This resulted in a total of 73 “initial” memories being recalled and dated at later time points, on average 2.28 memories per child (16 by the youngest group, 26 by the middle group, and 31 by the oldest group, whereby the youngest group recalled fewer initial memories than did the two older groups at marginal significance, *F*(2,29) = 2.94, *p* = 0.07, $\eta_{p}^{2}$ = 0.17). At the 8-year interview, 30 out of the 37 children (81.1%; six from the youngest group, 12 from the middle group, and 12 from the oldest group) recalled and dated at least one “new” memory that they produced 6 years ago at the 2-year interview. This resulted in a total of 55 “new” memories being recalled and dated at the two follow-up interviews, on average 1.83 memories per child (10 by the youngest group, 24 by the middle group, and 21 by the oldest group, whereby the mean number did not differ significantly across groups, *F*(2,27) = 0.51, *p* = 0.61, $\eta_{p}^{2}$ = 0.04). The “initial” memories (*M* = 42.96 months, *SD* = 21.60) were significantly earlier than the “new” memories (*M* = 55.69 months, *SD* = 22.30) at the first time when they were recalled, *F*(1,120) = 12.67, *p* = 0.0005, $\eta_{p}^{2}$ = 0.08.

Subsequent analyses focused on the age estimates of the “initial” and “new” memories at different time points. Preliminary analyses showed no systematic gender differences, so gender was not considered further. The variability in the timing of follow-up interviews across children did not affect the pattern of results. In line with our previous findings (Wang and Peterson, 2014), spontaneous (24%) and cued memories (76%) showed identical patterns and were pooled together in analysis. Given the small sample size, we included results with *p-*values close to 0.10. We emphasize the importance of considering effect sizes to appraise the strength of the evidence, which, unlike *p-*values, are not subject to the influence of sample sizes (Rosenthal and Rosnow, 1991).

### Initial Memories

We examined the age estimates of the initial memories across the three time points, with memory as the unit of analysis. Based on prior findings that memory events that occurred before 48 months were particularly prone to postdating (Wang et al., 2010; Wang and Peterson, 2014), we examined children’s memories initially dated before (52%) and after (48%) 48 months separately. We conducted a 3 (age group) × 3 (time point) × 2 (initial memory age: before or after 48 months) mixed model analysis on age estimates using SAS PROC MIXED program (Singer, 1998), with age group being a between-subject factor, time point and initial memory age being within-subject factors, and subject being a random factor. There was no significant 3-way interaction (*p* = 0.97), which was then excluded from the final model.

**FIGURE 1:**
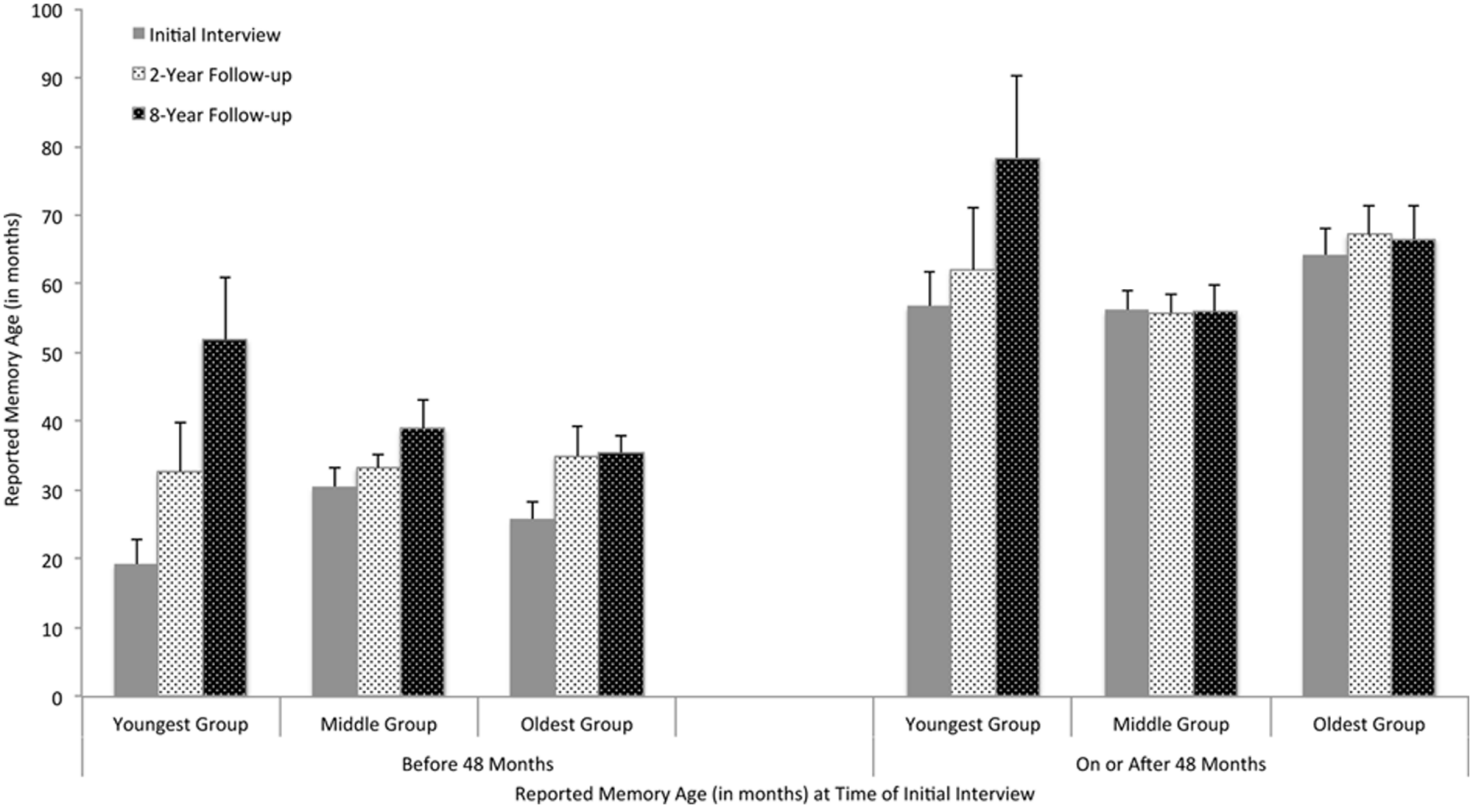
**Age of “initial” earliest memories dated at three time points as a function of age group and initial memory age.** Error bars represent standard errors of the means.

There were main effects of time point, *F*(2,151) = 14.81, *p* < 0.0001, Δ*R*^2^ = 0.19, and initial memory age, *F*(1,151) = 89.59, *p* < 0.0001, Δ*R*^2^ = 0.29, qualified by an Age group × Time, *F*(4,151) = 3.58, *p* = 0.008, Δ*R*^2^ = 0.06, and an Age group × Initial memory age interaction, *F*(2,151) = 2.95, *p* = 0.057, Δ*R*^2^ = 0.03. Further analyses were conducted with memories from before and after 48 months, separately. As shown in **Figure 1**, across all age groups, memories occurring before 48 months were generally postdated at the follow-up interviews, *F*(2,70) = 13.70, *p* < 0.0001, Δ*R*^2^ = 0.31. This was particularly true for the youngest group, *F*(2,21) = 7.91, *p* = 0.003, Δ*R*^2^ = 0.26, relative to the middle group, *F*(2,27) = 2.15, *p* = 0.14, Δ*R*^2^ = 0.05, or the oldest group, *F*(2,22) = 5.35, *p* = 0.01, Δ*R*^2^ = 0.26. Memories occurring after 48 months also showed an effect of time point, *F*(2,69) = 3.19, *p* = 0.05, Δ*R*^2^ = 0.04, which appeared to be driven solely by the youngest group who tended to postdate memories over time, *F*(2,4) = 4.03, *p* = 0.11, Δ*R*^2^ = 0.48.

To further test the effects of the initial memory age and the initial child age on the magnitude of postdating, we conducted regression analyses with memory age and child age at the initial interview (both being continuous variables) as predictors and the *change* in memory age at a subsequent interview (i.e., age estimates at the 2- or 8-year interview – age estimates at the initial interview) as the outcome variable, including subject in the models as a random factor. The initial memory age, *t* = –2.47, *B* = –0.35, *p* = 0.02, and the initial child age, *t* = –2.00, *B* = –0.33, *p* = 0.05, both negatively predicted the magnitude of postdating at the 8-year interview. A similar but non-significant trend also appeared at the 2-year interview for both memory age, *t* = –1.45, *B* = –0.12, *p* = 0.15, and child age, *t* = –1.31, *B* = –0.14, *p* = 0.20. Thus, confirming the findings from the mixed model analysis, earlier memories were postdated to a greater extent than later memories regardless of children’s age, especially as time further elapsed. As well, younger children postdated their memories to a greater extent than did older children, after an extended interval period.

In summary, childhood memories, especially those from earlier years and those of younger children, were subject to postdating over time. As a result, the average age of the very first memories children recalled increased from 35.81 months at the initial interview to 39.96 months 2 years later and to 52.54 months 8 years later. Thus, the boundary of childhood amnesia shifted substantially forward in time over the course of development. This may partially explain why younger children tend to provide earlier childhood memories than older children and adults (Peterson et al., 2005; Jack et al., 2009; Tustin and Hayne, 2010). It is important to further stress that the actual events being recalled by the children did not shift forward in time – the same events were recalled across the interviews – only children’s dating of them. In other words, the shift forward of the boundary of childhood amnesia is at least in part an artifact of systematic changes in memory dating.

Given that memories from the earlier years of life and those of preschool children are often retained with lesser quality and coherence than more recent memories and memories of older children and adults (Bauer, 2007; Pathman and Ghetti, 2014), they were particularly vulnerable to dating errors, consistent with previous findings (Friedman, 2005; Wang et al., 2010; Pathman et al., 2013; Wang and Peterson, 2014). In contrast, the memory age estimates by older children and of later memories seemed to be stabilized over time. This may reflect better memory retention and thus less postdating among older children and for more recent memories. In addition, as children grow older, dating information of earliest memories may be encoded as part of their memory or personal knowledge, which then remains stable thereafter.

### New Memories

Next, we examined the age estimates of the new memories first recalled and dated at the 2-year interview and again at the 8-year interview, with memory as the unit of analysis. Memories first dated before (29%) and after 48 months (71%) were examined separately. We conducted a 3 (age group) × 2 (time point) × 2 (initial memory age: before or after 48 months) mixed model analysis on age estimates using SAS PROC MIXED program (Singer, 1998), with age group being a between-subject factor, time point and initial memory age being within-subject factors, and subject being a random factor. The 3-way interaction was not significant (*p* = 0.91) and then excluded from the final model.

**FIGURE 2:**
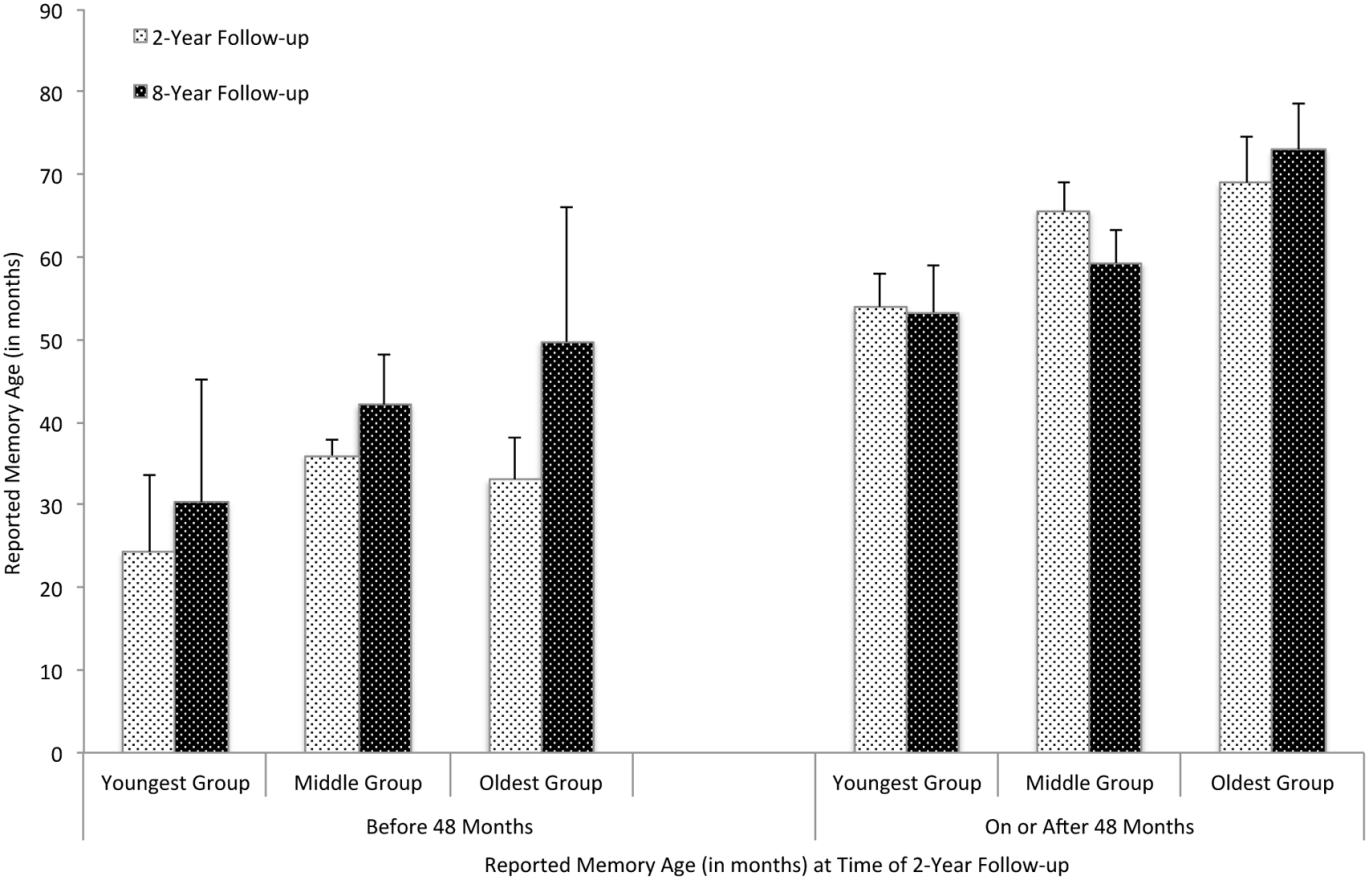
**Age of “new” earliest memories dated at the 2-year and 8-year interviews as a function of age group and initial memory age.** Error bars represent standard errors of the means.

A main effect of initial memory age emerged, *F*(1,73) = 37.36, *p* < 0.0001, Δ*R*^2^ = 0.37, qualified by a Time x Initial memory age interaction, *F*(1,73) = 4.23, *p* = 0.04, Δ*R*^2^ = 0.04. As shown in **Figure 2**, across all age groups, memories occurring before 48 months tended to be postdated between the 2-year and 8-year interviews, *F*(1,15) = 2.80, *p* = 0.12, Δ*R*^2^ = 0.07, whereas memories from after 48 months were not postdated, *F*(1,47) = 0.14, *p* = 0.71, Δ*R*^2^ = 0.02. As a result, the age differences between memories before and after 48 months decreased by the 8-year interview.

We further conducted a regression analysis to test the effects of memory age and child age at the 2-year interview on the *change* in memory age by the 8-year interview (i.e., age estimates at the 8-year interview – age estimates at the 2-year interview), including subject in the model as a random factor. Memory age at the 2-year interview negatively predicted the magnitude of postdating at the 8-year interview, *t* = –2.76, *B* = –0.27, *p* = 0.008. Thus, earlier new memories were postdated to a greater extent than later new memories as time went by, independent of children’s age. Children’s age was not a significant predictor of the magnitude of postdating.

Thus, following a 6-year interval, children’s memories were postdated such that the average age of the earliest new memories children recalled shifted later in time, from 49.57 to 54.90 months. Like the initial memories, earlier new memories were particularly prone to postdating whereas later memories remained relatively stable in age estimates over time. Interestingly, there was no age difference among children in the magnitude of postdating for new memories. Because new memories were considerably older than initial memories and were first recalled at the 2-year follow-up when children were all in their middle childhood or beyond, children of different age groups might not differ in their levels of retention (Bauer, 2007; Wang et al., 2014) and therefore showed similar levels of postdating.

## General Discussion

This prospective study investigated children’s recall and dating of earliest childhood memories at multiple time points over an extended period of time. In spite of the small sample, the effect sizes were comparable with previous studies (Peterson et al., 2011; Wang and Peterson, 2014). The cross-sectional longitudinal design allowed us to simultaneously examine the effects of age at encoding, retention interval, and age of children on memory dating. The findings showed that although children continued to remember many of the memories they recalled 8 years ago, they postdated the memories, especially the earlier ones, to considerably later ages as time passed. The memory age estimates seemed to be stabilized among older children and for older memories. The pattern of findings is consistent with both memories recalled at the initial interview (i.e., initial memories) and those newly recalled at the 2-year interview (i.e., new memories). The study further extends Wang and Peterson’s (2014) findings by showing that earliest memories *continued* to be postdated many years following the previous recalls and that the magnitude of postdating was smaller for older children and older memories. Perhaps over the course of development, the age estimates may eventually be integrated as part of the memory or personal “knowledge” so that later in retrospect we all “know” when our earliest memories took place.

We would like to emphasize our key finding: that young children *continued* the process of re-dating their memories for several years after the recalled events actually occurred. By the time children were 8 years older than initially, the age of estimated event occurrence was *more than a year* later. The magnitude of this re-dating is astonishing. This suggests that our accepted knowledge and wisdom (and our textbooks) may be wrong. If the average age of earliest memory identified in current research is 3.5 years and there is systematic mis-dating by a year or more, then people’s earliest memories may actually date from when they were 2-year-olds.

Note that we do not assume that memories were dated with absolute accuracy at the initial interview. It is the postdating of the same memories over time that is of interest. Indeed, children might have already made telescoping errors the first time they were interviewed for the memories. As shown in Wang et al. (2010), children postdated early memories compared with their parents, and adult studies have shown that memories from the beginning of a life period (e.g., childhood as in the current study) tend to show telescoping errors of postdating (Loftus and Marburger, 1983; Rubin and Baddeley, 1989). If the children in the current study were already making telescoping errors from the start, the magnitude of actual memory dating errors might be even larger than what we observed at the follow-up interviews. In addition, it is unlikely that children’s age estimates became more accurate over time, given that dating accuracy declines with retention interval in both children and adults (Janssen et al., 2006; Friedman et al., 2011).

## Conclusion

The present study spanned across 8 years. It yielded critical findings about the fate of early childhood memories, which have far-reaching implications. Again, we emphasize that the time of occurrence of the events being recalled by the children in this study did not shift forward in time. Rather, children’s dating of those memories shifted. Thus, as we suggested before (Wang and Peterson, 2014), people’s earliest memories may be earlier than they think. Prior reviews of the childhood amnesia literature have suggested that the average age of earliest memories among Western Europeans and North Americans is 3.5 years of age (e.g., Rubin, 2000). We suggest that the average age of earliest memories is probably earlier than that, and that distortions in memory dating may have led to erroneous conclusions about when our earliest memories occurred.

## Author Contributions

QW analyzed the data and drafted the manuscript. CP designed the study, supervised data collection and worked on the manuscript.

## Conflict of Interest Statement

The authors declare that the research was conducted in the absence of any commercial or financial relationships that could be construed as a potential conflict of interest.
